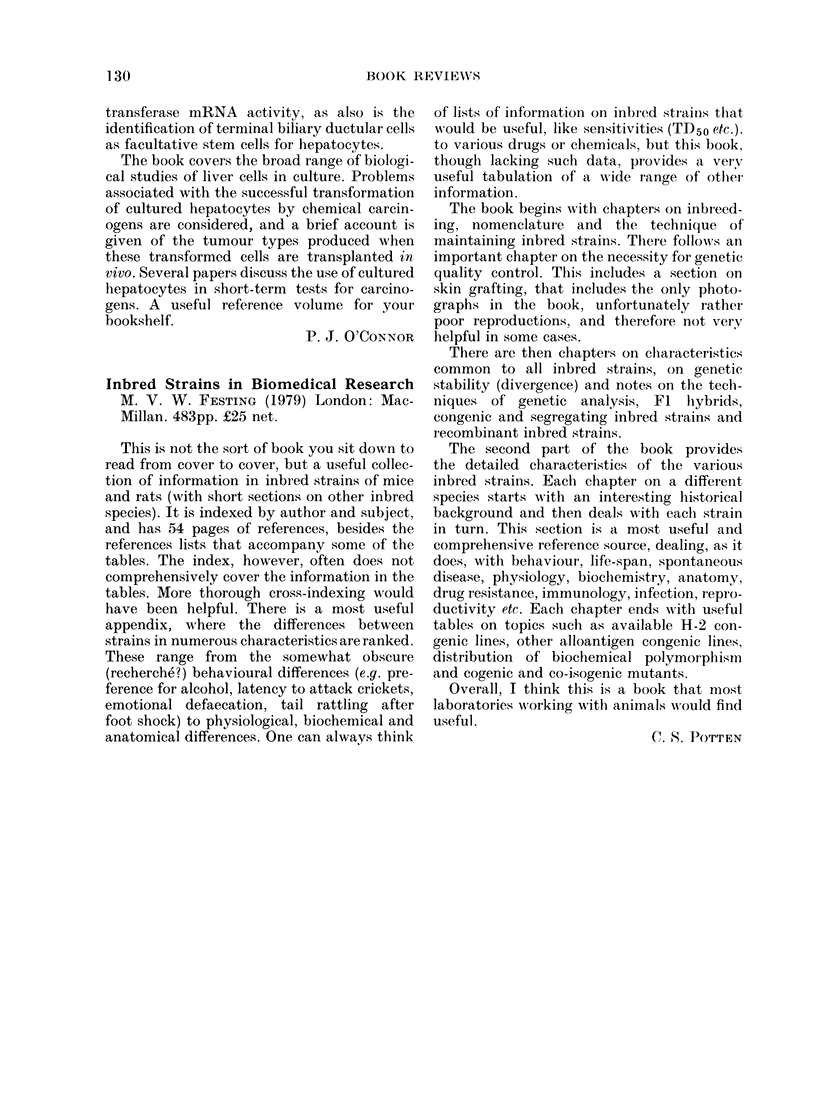# Inbred Strains in Biomedical Research

**Published:** 1981-07

**Authors:** C. S. Potten


**Inbred Strains in Biomedical Research**
M. V. W. FESTING (1979) London: MacMillan. 483pp. £25 net.

This is not the sort of book you sit down to read from cover to cover, but a useful collection of information in inbred strains of mice and rats (with short sections on other inbred species). It is indexed by author and subject, and has 54 pages of references, besides the references lists that accompany some of the tables. The index, however, often does not comprehensively cover the information in the tables. More thorough cross-indexing would have been helpful. There is a most useful appendix, where the differences between strains in numerous characteristics are ranked. These range from the somewhat obscure (recherché?) behavioural differences (*e.g.* preference for alcohol, latency to attack crickets, emotional defaecation, tail rattling after foot shock) to physiological, biochemical and anatomical differences. One can always think of lists of information on inbred strains that would be useful, like sensitivities ($TD_{50}$ *etc.*). to various drugs or chemicals, but this book, though lacking such data, provides a very useful tabulation of a wide range of other information.

The book begins with chapters on inbreeding, nomenclature and the technique of maintaining inbred strains. There follows an important chapter on the necessity for genetic quality control. This includes a section on skin grafting, that includes the only photographs in the book, unfortunately rather poor reproductions, and therefore not very helpful in some cases.

There are then chapters on characteristics common to all inbred strains, on genetic stability (divergence) and notes on the techniques of genetic analysis, F1 hybrids, congenic and segregating inbred strains and recombinant inbred strains.

The second part of the book provides the detailed characteristics of the various inbred strains. Each chapter on a different species starts with an interesting historical background and then deals with each strain in turn. This section is a most useful and comprehensive reference source, dealing, as it does, with behaviour, life-span, spontaneous disease, physiology, biochemistry, anatomy, drug resistance, immunology, infection, reproductivity *etc.* Each chapter ends with useful tables on topics such as available H-2 congenic lines, other alloantigen congenic lines, distribution of biochemical polymorphism and cogenic and co-isogenic mutants.

Overall, I think this is a book that most laboratories working with animals would find useful.

C. S. POTTEN